# Assessing Osteopathic Medical Students’ Perceptions on Training to Identify Social Determinants of Health: A Pilot Study in a Detroit Street Medicine Program

**DOI:** 10.51894/001c.144191

**Published:** 2025-09-30

**Authors:** Nikila Nallabelli

**Affiliations:** 1 College of Osteopathic Medicine Michigan State University

## 51

### INTRODUCTION

Basic needs like food, shelter, and clothing often take priority over healthcare for people experiencing homelessness (PEH). Understanding Social Determinants of Health (SDoH) is essential for future physicians to serve this population better. Detroit Street Care (DSC), a street medicine program at MSUCOM, provides osteopathic medical students (OMS) with hands-on experience in underserved communities. Previous data showed that 65.7% of PEH faced transportation barriers to accessing DSC’s free healthcare. This study explores the impact of training OMS to interact with and survey PEH at DSC.

### HYPOTHESIS/OBJECTIVES

We hypothesized that training OMS to survey PEH would enhance recognition of SDoH, particularly transportation barriers. Our goal is to evaluate the impact of unbiased, empathetic, and patient-centered training on OMS’s ability to collect and analyze data.

### METHODS

This cross-sectional study (IRB-STUDY00010091) evaluated trained OMS (N=18), surveying and collecting data from PEH regarding transportation barriers. All participants completed human research ethics training emphasizing humanistic interactions with PEH from the same urban area of Detroit-MI, where DSC operates. For objective/quantitative analysis (Chi-squared-CI95), we assessed OMS’s evaluation of PEH’s demographics, transportation needs, and health status compared to those who interacted with PEH for clinical purposes but didn’t administer the surveys. Testimonials were collected for subjective/qualitative analysis.

### RESULTS

Among trained OMS who saw patients, 50% responded, with 44.44% conducting interviews (P<0.05). One-third (33.33%) had prior experience with PEH, while 55.55% reported a changed perspective on transportation barriers after reviewing previous DSC-collected data. All (100%) identified transportation as a major obstacle to care.

### CONCLUSIONS

All students recognized transportation as a significant SDoH affecting PEH’s healthcare access. However, training alone was insufficient; direct interaction deepened understanding. Participants noted the need for hands-on engagement to address healthcare disparities. Despite the limited sample size, this pilot study suggests integrating field-based learning enhances medical training, fostering compassionate, well-rounded physicians to amplify the voices of PEH, where they are often overlooked. Expanding DSC’s experiential learning opportunities may equip OMS with skills to mitigate healthcare barriers and improve patient outcomes.

